# Learning the Structural Diversity of Olfactory Receptors: A Genomic Case Study in Two Longhorn Beetles (Cerambycidae: Lamiinae)

**DOI:** 10.3390/insects17060587

**Published:** 2026-06-04

**Authors:** Mataya Duncan, Terrence Sylvester, Emilee Walden, Jenniffer Roa Lozano, Emma Turner, Samuel Duncan, Robert F. Mitchell, Duane D. McKenna, Rich Adams

**Affiliations:** 1 Department of Entomology and Plant Pathology, University of Arkansas, Fayetteville, AR 72701, USA; 2 Center for Agricultural Data Analytics, University of Arkansas, Fayetteville, AR 72701, USA; 3 Department of Biological Sciences, University of Memphis, Memphis, TN 38152, USA; 4 Center for Biological Diversity, University of Memphis, Memphis, TN 38152, USA; 5 Department of Entomology, The Pennsylvania State University, University Park, PA 16802, USA; 6 Center for Chemical Ecology, The Huck Institutes of the Life Sciences, The Pennsylvania State University, University Park, PA 16802, USA

**Keywords:** protein prediction, olfaction, non-model genomics, AlphaFold, molecular evolution, unsupervised clustering

## Abstract

Recent advances in machine learning are transforming biological research by offering powerful tools to address complex challenges across the life sciences. In particular, deep learning approaches now enable accurate predictions of protein structure and function, opening new avenues for investigating proteomic diversity in non-model organisms. In this study, we conducted a genomic case study that examines the predicted structure and diversity of odorant receptor (OR) proteins in two species of longhorn beetles (Cerambycidae) with divergent life histories: the highly specialized red milkweed beetle (*Tetraopes tetrophthalmus*) and the broadly polyphagous Asian longhorned beetle (*Anoplophora glabripennis*). Using leading predictive algorithms, we inferred the structure of beetle-encoded OR genes, compared confidence scores, and assessed protein diversity across OR families and between the two genomes. Unsupervised clustering applied to pairwise protein comparisons revealed an expected strong correlation between structure and sequence, while supporting the evolutionary classification of previously predicted OR groups and revealing new evidence of previously unrecognized OR subclusters. Notably, we identify specific proteins exhibiting substantial structural divergence despite relatively low sequence divergence with other paralogs, suggesting potential outliers subject to unusual evolutionary processes. These results highlight the utility of statistical learning for uncovering patterns of protein evolution and structural diversity in understudied insect genomes.

## Simple Summary

Understanding the breadth of proteins that exist in nature remains a grand challenge in the life sciences. New approaches for predicting the structure and function of proteins hold great promise in this endeavor, particularly for non-model organisms with limited resources and understudied protein repertoires. Here we conducted a genomic case study to explore the application of machine learning for studying the structure and diversity of olfactory proteins in two longhorn beetles with markedly different life histories. Our goal was to investigate how structure prediction works for these hard-to-study receptors and to see how variation exists within and between species. We find evidence of structure variation across different gene families, and differences among algorithms depending on annotation quality. Modeling structure and sequence divergence confirms relationships between the two distances, while highlighting potential outliers. These results showcase the promise of AI-based structure prediction, which can help reveal hidden biological diversity in organisms that lack extensive laboratory data, with potential value for understanding insect biology and biodiversity broadly.

## 1. Introduction

Determining the structure and function of proteins has long stood as one of biology’s grand challenges [1,2]. Historically, this required labor-intensive methods such as X-ray crystallography, nuclear magnetic resonance (NMR) spectroscopy, and cryo-electron microscopy [3,4,5]. These traditional methods are notoriously time-consuming and technically demanding [6], with success depending on factors like crystal lattice properties, protein stability, and conformational flexibility [7,8].

Recent progress in artificial intelligence (AI) has paved new paths in structural biology. Deep learning algorithms now predict the 3D structure of proteins from amino acid sequences alone [9,10,11], often within minutes. These innovations are reshaping our understanding of protein biology and evolution [12] and have been applied across a wide array of biological subdisciplines—including biochemistry [13], molecular biology [14], and human health [15]—as well as in specific investigations such as chromatin architecture [16] and the evolution of SARS-CoV-2 variants [17,18]. Notably, AI-based prediction models have been successfully applied to the entire human proteome [19], and, more recently, to the proteomes of major model organisms such as *Caenorhabditis elegans*, *Mus musculus,* and *Arabidopsis thaliana* [20]. These breakthroughs now rank among the most widely cited scientific achievements of the decade—for instance, the AlphaFold paper has garnered over 30,000 citations (Google Scholar, accessed February 2025; [19]).

AI models hold special promise for non-model organisms, where traditional biochemical approaches are often underutilized due to limited experimental infrastructure and resources [21,22,23]. In the absence of efficient computational approaches, our understanding of protein diversity across nature is likely to remain incomplete—especially for poorly characterized genomes in highly diverse lineages and non-model organisms [24,25]. Beetles (Coleoptera) are among the most megadiverse insect orders, playing a key evolutionary role in shaping terrestrial biodiversity over the past 300 million years [26]. In addition to their remarkable taxonomic diversity, beetles are notable for their wide array of fascinating life history traits [27,28], their agricultural relevance as both pollinators and pests [29,30], and, more recently, for their remarkable genomic diversity [31]. Despite an estimated ~1.5 million species worldwide [32], only a very few—such as *Tribolium castaneum*—are considered model organisms [33]. Importantly, this means that a vast diversity of proteins encoded within beetle genomes remains entirely unexplored. To date, only a handful of beetle protein structures have been resolved using traditional biochemical techniques, including proteins involved in bioluminescence [34] and antifreeze [35]. However, recent efforts to assemble and annotate a growing number of beetle genomes [36,37,38,39,40,41], have begun to reveal the molecular underpinnings of this ecologically, environmentally, and economically important insect group [31,42].

Chemosensation plays a profound role in shaping many dimensions of beetle biology [43]. Essential to the beetle sensory system are odorant receptors (ORs)—membrane-bound proteins that detect volatile chemical compounds in the environment [44]. Broadly, insect ORs mediate a wide array of biological functions, including mate finding [43] and recognition [45,46], host seeking [47,48,49], chemical communication [50], and special navigation [51]. ORs are some of the most diverse and expansive gene families documented in beetle genomes sequenced to-date [31,50,52]. In most insects, ORs function alongside a highly conserved co-receptor, Orco, forming a tetrameric receptor channel [53]. Orco, present in nearly all extant insects, is typically highly expressed in olfactory neurons [53,54]. To date, no complete OR structure has been experimentally solved for any beetle species. Moreover, given the tremendous diversity of beetle genes and genomes in nature, it is likely that protein structures will never be experimentally solved for the vast majority of beetle ORs that exist. AI-based protein algorithms therefore hold great promise for studying these biologically critical yet poorly characterized proteins.

We recently sequenced and assembled the genome of the red milkweed beetle (*Tetraopes tetrophthalmus*; Cerambycidae; [55]), uncovering a diverse array of OR genes in this highly host-specific insect [56,57,58]. As in many longhorn beetles, the species exhibits exaggerated antennae—a morphological hallmark of Cerambycidae, reflecting their well-developed chemosensory biology [28,59]. *T. tetrophthalmus* is a charismatic member of the herbivore community associated with North American milkweeds (*Asclepias* spp.) [60] and has been the focus of extensive research on coevolution [56,57], host plant specialization [61,62], population genetics [63], and chemical ecology [64].

Here we use the *T. tetrophthalmus* genome as a genomic case study to explore the methodological behavior of AI-based algorithms for learning OR protein structure and diversity in longhorn beetle genomes. To provide a comparative perspective, we also examine the predicted OR repertoire of the Asian longhorned beetle (*Anoplophora glabripennis*)—a globally invasive, highly polyphagous pest and the first cerambycid species with a sequenced genome [42,65]. These two species (*T. tetrophthalmus* and *A*. *glabripennis*) share a common ancestor ~70 MYA [66]. The contrasting life histories of these two species raise interesting questions relevant to our investigations. For example, recent hypotheses have suggested that host-specialized species may show expansions in OR groups involved in detecting host-specific cues, whereas relative generalists may maintain a more heterogeneous OR landscape, consistent with studies linking OR evolution to ecological adaptation and receptor tuning [67,68]. Likewise, species with relatively broad host ranges may exhibit greater OR diversity and lower levels of pseudogenization than specialists, reflecting selection for detecting chemically diverse environments [69,70]. Differences in evolutionary history may be associated with lineage-specific expansions or contractions of particular OR clades, potentially reflecting ecological shifts or divergence in host utilization over time [69,71,72].

Our study is primary focused on technical insights for learning protein structure and diversity based on predicted pairwise similarity metrics [73]. We applied unsupervised clustering with dimensionality reduction to characterize patterns of protein structural diversity within and between these two genomes and to examine the relationship between protein structure and sequence-based evolutionary divergence. Specifically, we asked: (1) How similar are OR protein structures predicted from two different algorithms and how do they react to incomplete protein sequences? (2) What is the extent of OR structural diversity in the two cerambycid genomes studied? (3) Do cross-species structural comparisons cluster more strongly by sequence similarity, species identity, or both? (4) How well do structural distances correlate with sequence-based evolutionary distances?

## 2. Materials and Methods

### 2.1. Protein Sequences

We obtained amino acid sequences for 122 OR genes from the curated annotation of the *T. tetrophthalmus* genome [55]. To enable cross-species comparisons, we also included 133 OR genes from the Asian longhorned beetle (*A. glabripennis*) genome [42], a species that is in the same cerambycid subfamily (Lamiinae) and which has been the subject of intensive chemosensory annotation efforts [44,50,65]. For clarity, ORs annotated in the *T. tetrophthalmus* genome are hereafter labeled as “TETRA,” while those identified in *A. glabripennis* are labeled as “AGLAB”.

Our primary analyses were focused on a set of high-quality OR gene models with complete annotations available for the entire, full-length protein-coding sequence. We refer to this set as the “complete” ORs, which provide the focal point for exploring comparative insights into the diversity of putatively functional ORs in both cerambycid species. In total we identified 73 complete ORs in the TETRA genome and 95 complete ORs in AGLAB (Table S1). These sequences span eight previously characterized OR subfamilies found throughout Coleoptera (Groups 1, 2A, 2B, 3, 4, 5A, 5B, and 7; [50,74,75]).

Separately, we recovered a set of “incomplete” ORs in both TETRA (49) and AGLAB (38) with low-to-poor quality annotations with missing exons, premature stop codons or other irregularities (Tables S1 and S2). We emphasize that gene “completeness” is a continuum (Table S2), ranging from the absence of a single small exon to more extensive deletions of entire domains, which may result from biology (e.g., pseudogenization) or methodology (i.e., poor annotations). Annotation quality (or lack thereof) can be a persistent problem in undercharacterized, non-model insect genomes, and thus, we included incomplete genes in certain analyses as a simple technical comparison of predictive algorithms applied to variable annotation quality. Thus, we leveraged these incomplete ORs as an opportunity to gain technical insight into the behavior of algorithms when applied to recalcitrant gene models, whether due to annotation quality or real biological phenomena (e.g., pseudogenes). For the subset of analyses that include incomplete ORs, our rationale therefore focused on the comparison of the methodological behavior of algorithms on incomplete protein models. For example, is predicted confidence level lower or higher on incomplete ORs from AlphaFold versus RoseTTAFold? Detailed information regarding protein sequences, genomic context, and gene completeness is provided in the Supplement (Tables S1 and S2).

### 2.2. Protein Structure Predictions

We predicted structures for all 122 TETRA ORs using two deep learning models: AlphaFold version 2.3.2 [11] and RoseTTAFold version All-Atom (RFAA) [9]. Likewise, we also applied AlphaFold to predict the structures of all 133 AGLAB ORs for comparative analyses. We first compared these two algorithms to provide technical insight to predictions of beetle OR structures, while downstream analyses of OR structural and functional diversity were based on AlphaFold predictions [11,76,77,78]. AlphaFold models were generated using Google Colab Notebooks [79] according to the recommended default settings, including Amber energy minimization to obtain relaxed structures, as suggested in prior studies [10,80]. RoseTTAFold predictions were generated via the Robetta web server (https://robetta.bakerlab.org/ accessed on 5 March 2024), which implements the RoseTTAFold algorithm using default parameters. Each tool produced five structural models per protein; we retained the relaxed model with the highest confidence score for each OR, following established best practices [77]. Final protein models were saved in PDB format for downstream analysis.

### 2.3. Comparing AlphaFold and RoseTTAFold

We first evaluated consistency between AlphaFold and RoseTTAFold to provide technical insights and better understand their predictive properties on both complete and incomplete OR sets. Specifically, we obtained and compared the top-scoring relaxed models for each TETRA OR from both methods. Pairwise structural distances among predicted OR structures were quantified using root mean squared deviation (RMSD), both with and without a 15 Å cutoff—an approach commonly used to mitigate potential alignment outliers [81,82]. Alignments and RMSD calculations were performed using ChimeraX’s ‘Matchmaker’ function [18,83]. Confidence scores were extracted from the prediction output files for both AlphaFold and RoseTTAFold, and a delta score ($\Delta_{score}$) was computed to contrast confidence between algorithms:

$$\Delta_{score}=\left({AlphaFold}_{score}-{RoseTTAFold}_{score}\right)$$

Positive $\Delta_{score}$ values indicate higher confidence from AlphaFold, and negative values indicate higher confidence from RoseTTAFold. To compare the two scores on a similar scale, we used the normalized ${AlphaFold}_{score}$ and ${RoseTTAFold}_{score}$ that are both bounded by zero and one, representing little (near zero) to high confidence (near one), with moderate levels of confidence in-between. These two scores provided only relative predictive confidence in the global structure, and thus do not quantify accuracy, nor do they measure the true biological correctness of a predicted structure. Therefore, we included $\Delta_{score}$ only as a technical comparison of relative algorithm confidence because the true structures of these beetle ORs have not been experimentally solved. Specifically, we compared algorithm confidence between predictions derived from complete versus incomplete ORs, with the *a priori* expectation that both methods should return lower confidence on incomplete ORs due to their fragmented nature.

### 2.4. Phylogenetic Trees

We obtained a phylogeny of all 255 OR genes (122 TETRA and 133 AGLAB) from the original TETRA genome study [55]. Briefly, this tree was inferred using FastTree 2.1 [84] under the JTT substitution model with gamma-distributed rate variation and branch support assessed via Shimodaira–Hasegawa tests. Prior to phylogenetic construction, the OR protein sequences were aligned using Clustal Omega 1.2.4 [85], and processed with trimAl [86] (similarity threshold = 0, gap threshold = 0.8, conservation cutoff = 25). This produced a phylogeny with branch lengths representing evolutionary distance in units of expected amino acid substitutions per site. From this full phylogeny, four pruned subtrees were generated for focused analyses: (1) all ORs (complete + incomplete) from both species (255 total), (2) only complete genes from both species (168 total), (3) all TETRA ORs (122 total), and (4) only complete TETRA ORs (73 total). Pairwise sequence-based evolutionary distances were derived from phylogenetic branch lengths using the *cophenetic.phylo* function in the R package Ape 5.8 [87,88].

### 2.5. Unsupervised Learning of OR Protein Structural Diversity Within TETRA

We assessed OR structural diversity in TETRA by calculating RMSD for all pairwise combinations for AlphaFold-predicted structures (7381 comparisons), using the recommended 15 Å cutoff in ChimeraX 1.7. This yielded a structural distance matrix, which we paired with a corresponding matrix of sequence-based evolutionary distances extracted from the TETRA phylogeny described above. Separately, we pruned these datasets to include only the subset of 73 TETRA ORs with complete protein sequences to provide a comparison of analyses with and without incomplete genes; this provided a structural distance matrix comprising 2628 comparisons, which we paired with a matched sequence-based distance matrix from the pruned TETRA phylogeny.

We applied t-distributed Stochastic Neighbor Embedding (t-SNE; [89]) to each matrix to investigate patterns in structural and evolutionary divergence. t-SNE is a non-linear dimensionality reduction technique that seeks a lower-dimensional representation while preserving local relationships and structure in the data. This approach has been applied successfully in a number of applications for exploring and visualizing large-scale pairwise distance matrices [90,91,92,93]. Here we utilize t-SNE as an exploratory tool for compressing high-dimensional protein distances to discover visual patterns and to generate hypotheses. That is, we leveraged this approach to explore the high-dimensional space and uncover interesting and potentially overlooked patterns hidden within the protein comparisons. We applied the *Rtsne* function provided in Rtsne package 0.17 [94] according to the recommended default settings with Barnes-Hut implementation.

### 2.6. Cross-Species Comparisons of OR Structural and Sequence Diversity

To compare OR protein diversity across species, we first analyzed all 255 AlphaFold-predicted ORs from TETRA and AGLAB including all complete and incomplete genes. Pairwise RMSD comparisons (32,385 total) were used to construct a cross-species structural distance matrix. In parallel, we obtained sequence-based distances matched to the same pairwise comparisons from the corresponding phylogeny. To provide a comparison of analyses with versus without incomplete genes, we pruned these datasets to only include the 168 complete TETRA and AGLAB ORs (14,028 total). As before, we applied t-SNE to each of the pairwise distance matrices to explore and visualize the high-dimensional space in a lower-dimensional representation. Lastly, we plotted protein structural-based distances as a function of sequence-based (evolutionary) distances to assess evidence of the relationship between the two measures of protein divergence. For these analyses, we computed Pearson correlation coefficients (*r*) as only a descriptive measure between protein and sequence distance across all comparisons within each of the two species, as well as a combined analysis containing all ORs across both species together. Additionally, we conducted a Mantel test to evaluate whether structural and sequenced based distances are correlated. Specifically, we used the mantel function provided in the R package vegan 2.6-6 [95] with default settings and 1000 bootstrap replicates. We focused our analyses and visualization on the comparisons using complete OR proteins.

## 3. Results

### 3.1. Predicting the Structure of Beetle OR Proteins

**Figure 1:**
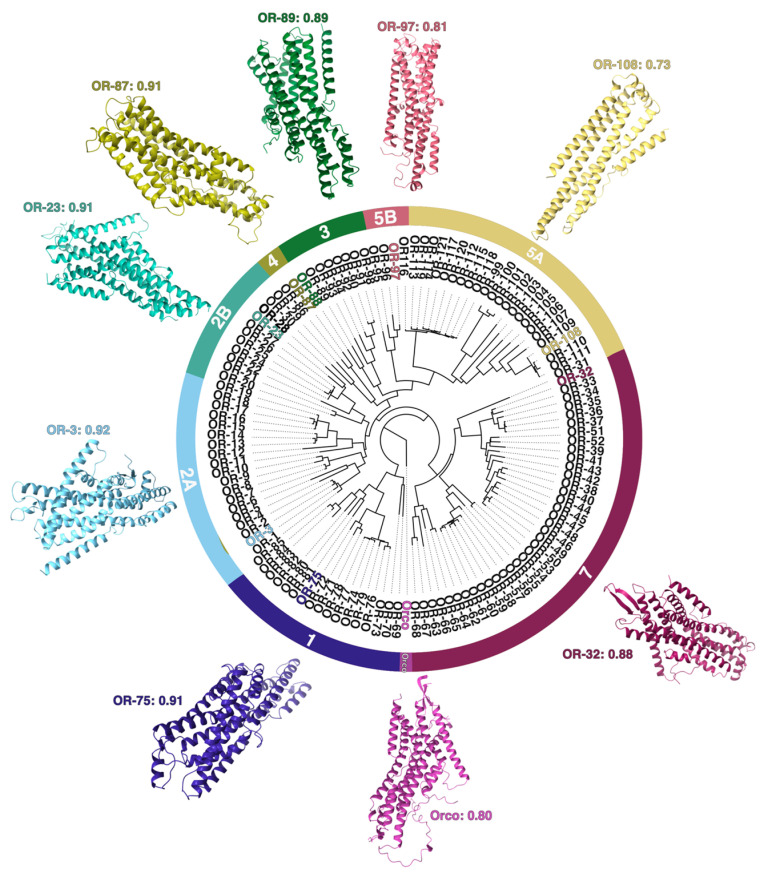
Exploring OR proteins in the *Tetraopes tetrophthalmus* (TETRA) genome. Shown are predicted structures for specific ORs with the highest normalized AlphaFold confidence score for each of the nine OR groups. Labels for each structure indicate the specific OR and its associated confidence score.

**Figure 2:**
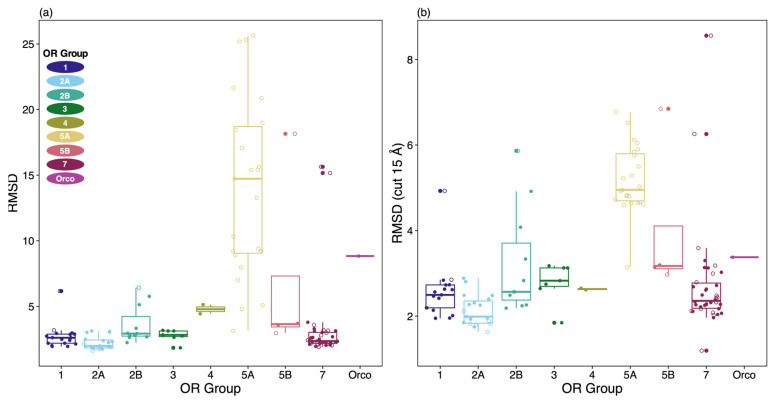
Comparing AlphaFold versus RoseTTAFold structural predictions for individual proteins. RSMD (no cutoff) distributions between predicted models shown as boxplots for each OR group in (**a**). Likewise, distributions of RMSD with a 15 Å cut are shown as boxplots in (**b**). Filled shapes represent complete ORs, while hollow shapes indicate incomplete ORs.

**Figure 3:**
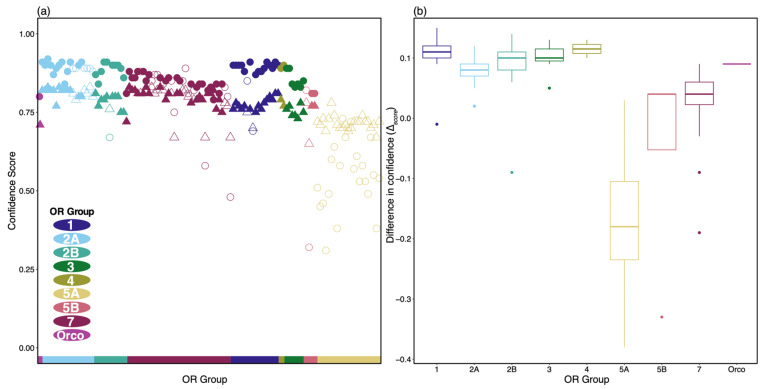
Comparing AlphaFold and RoseTTAFold confidence scores across TETRA ORs. Panel (**a**) shows individual confidence scores returned from both algorithms for each OR, while panel (**b**) provides boxplots of $\Delta_{score}$ from AlphaFold versus RoseTTAfold. Colors coordinate with OR groups, while circles indicate AlphaFold predictions and triangles represent RoseTTAFold predictions, respectively. Filled shapes represent complete ORs, and hollow shapes denote incomplete ORs.

We predicted structures for all 122 OR proteins from the TETRA genome using both AlphaFold and RoseTTAFold. As an initial overview, the highest-confidence AlphaFold model from each OR group was visualized alongside the full TETRA OR phylogeny (Figure 1). These representative structures had AlphaFold confidence scores ranging from 0.73 to 0.92 (mean: 0.86; scale: 0–1). Comparative analysis of AlphaFold and RoseTTAFold predictions revealed both similarities and differences in structural outputs (Figure 2 and Figure 3). Pairwise RMSD values computed with and without a 15 Å cutoff showed broadly consistent patterns (Figure 2a vs. Figure 2b). Across all 122 ORs, the mean RMSD between AlphaFold and RoseTTAFold structures was 5.2 Å without the cutoff and 3.2 Å with it. Restricting the analysis to only complete ORs reduced the mean RMSD to 2.79 Å (no cutoff) and 2.54 Å (with cutoff).

Variation in RMSD differed by OR group and gene completeness. For instance, Group 5A, which consisted entirely of incomplete genes in TETRA, showed high structural variability and the highest mean RMSD between AlphaFold and RoseTTAFold (average: 14.0 Å; Figure 2a). In contrast, groups with a higher proportion of complete genes—such as Group 1 (2.73 Å), Group 2A (2.22 Å), Group 2B (3.61 Å), Group 3 (2.79 Å), and Group 7 (3.20 Å)—exhibited much lower structural divergence between the two algorithms. A few notable outliers with large RMSD values were observed, particularly among incomplete Group 7 proteins (Figure 2). Indeed, pairwise distances for the same incomplete OR but different algorithm (AlphaFold versus RoseTTAFold for the same OR) spanned the entire range of RMSD observed (discussed below).

AlphaFold yielded higher relative confidence scores than RoseTTAFold for 79% of TETRA ORs (96 out of 122; Figure 3a). This percentage rose to 98% (72 of 73) when analysis was limited to only complete ORs. Delta confidence scores ($\Delta_{score}$) were generally positive across most OR groups (Figure 3b) with the exception of TETRA Group 5A—comprised exclusively of incomplete genes—where RoseTTAFold provided consistently higher relative confidence, reflecting by negative $\Delta_{score}$. On average, AlphaFold confidence scores exceeded those of RoseTTAFold by 2% across all ORs, and by 7% when restricted to only complete sequences. Notably, AlphaFold confidence scores consistently approached or exceeded 0.80 for complete proteins, in line with previous studies on other protein families [96].

### 3.2. OR Structural Diversity Within the TETRA Genome

**Figure 4:**
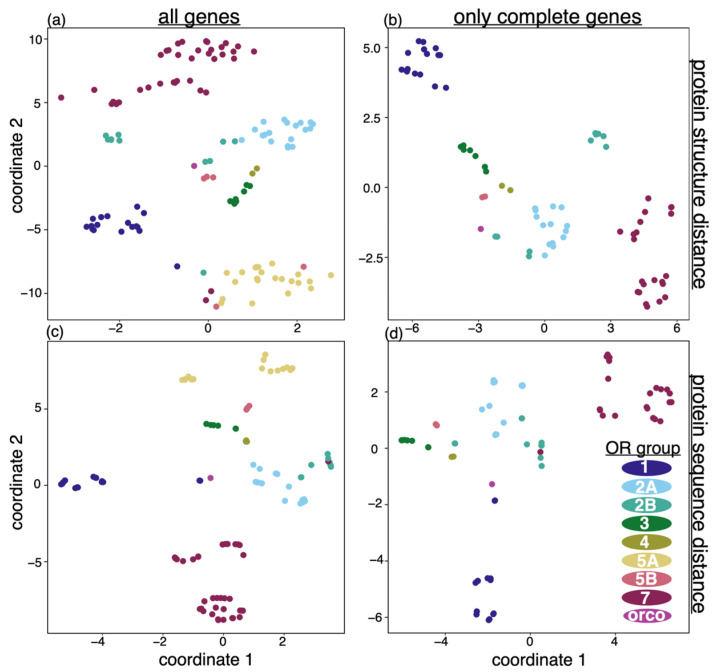
Unsupervised learning with t-SNE for OR proteins encoded in the TETRA genome. Colors are based on previously assigned OR group designation. Results are shown for t-SNE based on structural distances for analyses containing all genes (**a**) and only complete genes (**b**), and likewise for analyses based on phylogenetic distances for all ORs (**c**) and only complete ORs (**d**).

Dimensionality reduction with t-SNE revealed distinct patterns of OR structural and evolutionary diversity within the TETRA genome (Figure 4). Observed patterns based on pair-wise protein structural distances were largely congruent with previously defined OR groups. Broadly comparable clustering was observed when t-SNE was applied to both structural distances (top panels, Figure 4) and sequence-based distance inferred from the phylogeny (bottom panels, Figure 4). Resolution improved substantially when analyses were restricted to only complete genes (Figure 4b vs. Figure 4a; Figure 4d vs. Figure 4c). For example, Groups 1, 2A, 3, and 7 formed well-separated clusters in both structural and sequence space. Within Group 7, evidence of two distinct subclusters emerged based on protein distances, suggesting potential subclade diversification within this group. Group 2B also became more clearly distinguishable when incomplete genes were excluded. Group 1 and Group 7 strongly diverged from one another, particularly in the structure-based analysis (Figure 4b).

### 3.3. OR Diversity Across the TETRA and AGLAB Genomes

**Figure 5:**
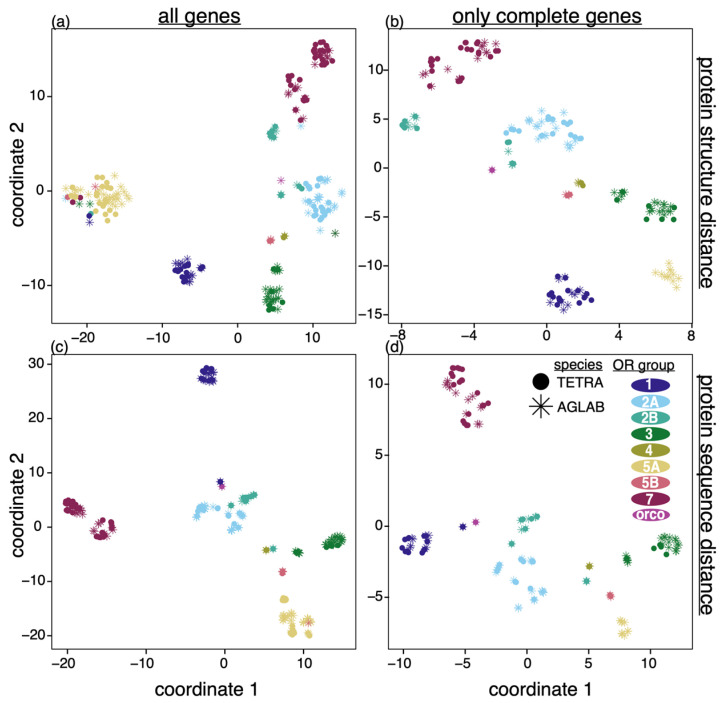
Exploring t-SNE analyses for OR proteins in both *Tetraopes tetrophthalmus* (TETRA, circle) and *Anoplophora glabripennis* (AGLAB, star) genomes. Colors indicate OR group assignments based on previous studies. Results shown for t-SNE based on structural distances for analyses containing all genes (**a**) and only complete genes (**b**), respectively, and likewise for analyses based on phylogenetic distances for all ORs (**c**) and only complete ORs (**d**).

Cross-genome comparisons of ORs encoded within TETRA and AGLAB further revealed patterns of structural diversity (Figure 5). As with the TETRA-only analyses (Figure 4), t-SNE revealed evidence of clear similarities to OR groups based on previous designations. Well-defined clusters were evident for Groups 1, 3, and 7 (Figure 5a), each containing representatives from both species (Figure 5). Similar clustering patterns emerged whether t-SNE was applied to sequence-based distances or protein structural distances (bottom vs. top rows; Figure 5). Exclusion of incomplete gene models sharpened cluster boundaries (right vs. left columns; Figure 5). For example, Groups 1, 2A, 2B, and 7 were far better resolved in structural space when only complete genes were analyzed (Figure 5c). Additionally, both structural and sequence-based analyses consistently supported the subdivision of Groups 3 and 7 into two distinct subclusters, each containing ORs from both beetle species. These patterns indicate parallel diversification within the same OR groups across the two species. Overall, t-SNE recovered patterns that were strongly organized by OR group, rather than species. That is, homologous ORs of both species tended to cluster together based on their previous designations.

### 3.4. Structural vs. Phylogenetic Distance

**Figure 6:**
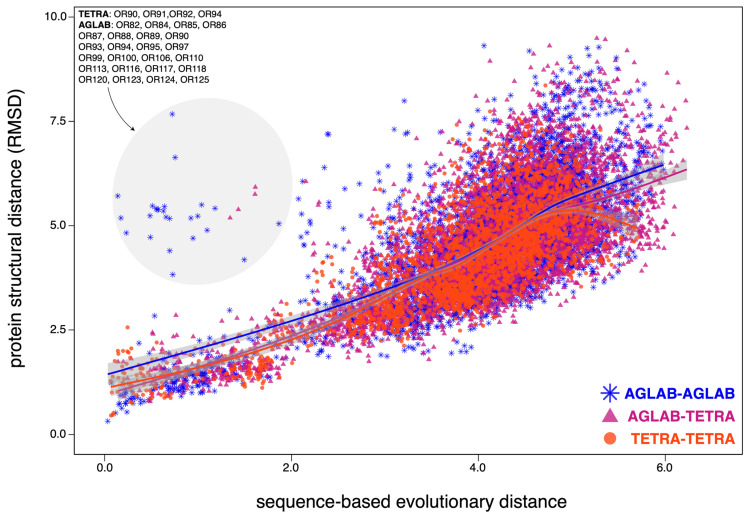
Relating structure-based and sequence-based distances for pairwise comparisons of only complete OR proteins within *Anoplophora glabripennis* (AGLAB, blue), within *Tetraopes tetrophthalmus* (TETRA, red), and between AGLAB and TETRA (purple). Curves and associated confidence intervals depict recovered relationships from non-parametric LOESS regression.

Comparisons of structural and sequence-based distances revealed strong positive correlations both within each species and between the two species (Figure 6). Within TETRA, the Pearson correlation coefficient (Pearson’s *r*) between structure and sequence distance was *r* = 0.76 when restricted to complete genes and slightly weaker at *r* = 0.62 when all incomplete genes were also included. A similar trend was observed for the full dataset of 255 ORs across both TETRA and ALGAB together (*r* = 0.72) when only complete genes were considered. Likewise, the mantel statistic was estimated to be 0.74 and significant with *p*-value = 0.001. Most pairwise comparisons clustered in the center of the distance ranges, reflecting a moderate to high level of divergence. Several pairwise comparisons were also identified as possible outliers from the bulk of these distributions as proteins with high structural divergence despite relatively low sequence divergence (left side of Figure 6). These outliers were primarily found in Group 5A of AGLAB and a handful of examples of Group 3 of TETRA, suggesting potentially unusual evolutionary trajectories specific to these ORs.

## 4. Discussion

Advances in statistical modeling are transforming our ability to study evolution and biodiversity broadly across the Tree of Life [12,13,14,15,16,97,98,99,100,101]. In this study, we applied supervised learning to predict protein structures, followed by unsupervised learning with t-SNE to investigate the structure and diversity of ORs—an evolutionarily dynamic and functionally diverse, yet under-characterized lineage of insect chemoreceptors. Our results contribute to a deeper understanding of OR structural diversity in beetles and contribute to broader efforts to understand proteins in non-model organisms [50,102,103].

As a result of rapid evolution and other processes, OR genes are notoriously challenging to annotate, and many remain functionally uncharacterized, particularly in non-model insects [50,104,105,106]. Efforts to better recover these important genes will be essential to provide a complete picture of olfactory evolution in beetles and other organisms. Thus, we primarily focused our analyses on the comparison of complete ORs with high-quality annotations spanning the entire length of the predicted protein. Yet, like many under characterized genomes, we nonetheless found a large set of poorly annotated, incomplete ORs, and only included these as a case study for several technical comparisons of predictive algorithms and t-SNE clustering. Both AlphaFold and RoseTTAFold generated viable structural predictions for beetle ORs, but the two algorithms reacted differently to gene completeness. Consistent with previous work [77,107], generally, we found evidence that structural predictions of identical OR sequences differed notably between the two methods, sometimes outpacing comparisons of different genes on the same algorithm. For example, the distance between AlphaFold and RoseTTAfold predictions for the same OR was often greater than the distance between two different ORs using AlphaFold alone. These data dovetail with previous work that has found AlphaFold to produce more accurate structural models [77], and these conclusions ultimately led us to base our downstream analyses on the AlphaFold structures.

Our study highlights the growing potential of machine learning to investigate protein structure and diversity in non-model organisms, underscoring its broad potential across diverse gene families and taxa. Our application of unsupervised learning revealed a high degree of OR structural diversity in both beetle species. The resulting t-SNE clusters were well-aligned with prior phylogenetic classifications based on sequence data alone, underscoring the potential of structural comparisons for investigating and characterizing gene family classifications [108,109]. Notably, our structural-based comparisons also identified two prominent subclusters within OR Groups 3 and 7, suggesting possible recent divergence or perhaps cryptic subfunctionalization within these lineages. Future studies will be necessary to uncover the evolutionary processes underlying driving these apparent patterns.

We also observed strong correlations between structural and sequence-based distances, reinforcing the close predicted relationship between these two dimensions of protein evolution (Figure 6). However, a subset of ORs—primarily within AGLAB Group 5A—displayed high structural divergence despite comparatively low sequence divergence. Such patterns may result from a diversity of factors, such as adaptive shifts, relaxed constraints, or other potential evolutionary processes. Group 5A is known to have a high degree of lineage specificity among the ORs, yet it is also considered to be one of the youngest of all the beetle OR subfamilies, only appearing with the Bostrichoidea [42]. Yet, we cannot yet rule out technical limitations in current AI-based prediction models for membrane-bound receptors. Future studies incorporating broader taxonomic sampling of beetle genomes will be essential to determine whether these patterns are conserved across taxa and evolutionary timescales. Additionally, investigating predicted ligand-binding sites and affinities [110,111,112]—especially within the subdivided groups—may shed light on the biological and evolutionary significance of the observed structural divergence and whether it reflects functional differences among beetle OR genes.

Despite encouraging results, several limitations remain, suggesting new paths for future work at the interface of OR structure, function, and evolution. ORs are membrane-bound proteins with complex architectures and often limited available structural data, making them particularly challenging to model [113,114]. Both AlphaFold and RoseTTAFold are known to underperform on membrane protein structures [19,115], and both struggle to account for conformational flexibility—an important factor for ORs that undergo dynamic changes upon binding to volatile ligands [52,53,116,117]. Nonetheless, these tools provide practical, scalable solutions for studying protein structure in data-poor systems, and future improvements in AI modeling are likely to address many of their current limitations [78].

Recent studies have increasingly leveraged AI to predict and identify ligand-binding sites [118,119,120], offering valuable insights into receptor function and molecular interactions. Such knowledge of OR structure and function can help guide informed and targeted strategies for biotechnical applications [121,122,123]. For example, synthetic compounds that mimic or block natural odorants can be designed to lure pests into traps or prevent them from locating crops [121,122,123]. Indeed, informed practices based on OR biology have been proposed as a key component for directing efforts to control the Asian longhorn beetle itself [65]. Moreover, OR studies have implications in biosensor development, where insect-derived receptors are used to detect volatile organic compounds in environmental monitoring [121,122,123]. Collectively, our study demonstrates the power of combining deep learning and unsupervised clustering to explore protein diversity and evolution, even in organisms with largely uncharacterized proteomes. By integrating structural and sequence-based analyses, we identify both conserved patterns and surprising outliers in OR evolution—laying the groundwork for future genomic, functional, and ecological investigations into insect chemosensory diversification.

## Data Availability

Protein sequences and associated data are available with the Supplementary Materials of this study, and the original published genomes [42,55].

## Supplementary Materials

The following supporting information can be downloaded at https://www.mdpi.com/article/10.3390/insects17060587/s1, Table S1: OR gene data; Table S2: Gene completeness data.
